# Clinical Risk Factors for Severe *Clostridium difficile*–associated Disease

**DOI:** 10.3201/eid1503.080312

**Published:** 2009-03

**Authors:** Timothy J. Henrich, Douglas Krakower, Asaf Bitton, Deborah S. Yokoe

**Affiliations:** Brigham and Women’s Hospital, Boston, Massachusetts, USA (T.J. Henrich, D. Krakower, A. Bitton, D.S. Yokoe); Massachusetts General Hospital, Boston (D. Krakower); Harvard Medical School, Boston (Deborah S. Yokoe)

**Keywords:** Clostridium infections, Clostridium difficile, colitis, risk factors, antibiotics, epidemiology, research

## Abstract

Identifying patients who are at high risk for severe *Clostridium difficile*–associated disease (CDAD) early in the course of their infection may help clinicians improve outcomes. Therefore, we compared clinical features associated with severe versus nonsevere CDAD by retrospectively reviewing records of hospitalized patients whose fecal assays were positive for *C. difficile* toxin. Of 336 patients, 12.2% had severe disease and 10.1% died from all causes. Regression modeling showed the following to be significantly associated with severe CDAD (p<0.05): age >70 years (odds ratio [OR] 3.35), maximum leukocyte count >20,000 cells/mL (OR 2.77), minimum albumin level <2.5 g/dL (OR 3.44), maximum creatinine level >2 mg/dL (OR 2.47), small bowel obstruction or ileus (OR 3.06), and computed tomography scan showing colorectal inflammation (OR 13.54). These clinical and laboratory markers for severe disease may be useful for identifying patients at risk for serious outcomes or death.

## Abstract 2

Rapidly available information, such as age and laboratory and radiologic data, can be used to identify adverse outcomes.

The incidence and severity of *Clostridium difficile*–associated disease (CDAD) is increasing in North America (*1*–*3*) and Europe (*4*,*5*). During the past 10 years in the United States, prevalence, case-fatality rates, total attributable mortality rates, and colectomy rates for persons with CDAD have markedly increased (*6*). Acquisition of *C. difficile* and the development of severe CDAD is associated primarily with healthcare, although community-acquired severe disease among persons previously thought to be at low risk for infection have been reported (*5*,*7*,*8*). Several mechanisms for increased disease severity have been proposed, including emergence of specific strains with genetic polymorphisms that encode higher levels of bacterial toxins A and B and the production of a binary toxin (*3*,*9**,**10*). The Centers for Disease Control and Prevention has reported outbreaks of CDAD associated with the new BI/NAP1 strain in 40 of 50 US states, although the association between BI/NAP1 and severe disease was not consistent among all facilities (*11*).

Host factors are also likely to be predictors of illness and death. For example, in an elderly population, leukocytosis, hypoalbuminemia, and nasogastric tube feedings were associated with high mortality rates from CDAD (*12*). Severity of underlying illness, as measured by an increased Horn score, has only moderate association with severe CDAD (*13*). Exposure to specific antimicrobial drugs, notably fluoroquinolones, clindamycin, and cephalosporins, has been associated with severe CDAD in some studies (*2*,*14*) but not others (*13*). Overall, previous studies have identified few clinical characteristics that consistently predict severe CDAD.

Identifying patients who are at high risk for severe CDAD early in the course of their infection might help clinicians improve patient outcomes, but predictors are not well known. Genetic subtyping, binary toxin assays, and culture of isolates are currently not widely accessible, which makes integrating knowledge of emerging bacterial factors into patient management difficult. To elucidate patient and clinical factors associated with severe CDAD, we conducted a 1-year retrospective study of Brigham and Women’s Hospital patients who had had positive *C. difficile* toxin results.

## Methods

### Study Population

We performed a retrospective chart review of electronic medical records for all patients who had had a positive fecal result for *C. difficile* toxin from June 2005 through May 2006, a period of increased incidence of severe CDAD in this hospital. During June–April 2006, CDAD was diagnosed by cytotoxic assay for cytopathic effects in cell culture. During May 2006, our laboratory changed to a toxin A and B ELISA. Before assay replacement, samples were tested with both techniques and results were comparable (A. Onderdonk, pers. comm., 2008).

All inpatients >18 years of age who had had a positive fecal *C. difficile* toxin result were included in the study. Ambulatory and emergency department patients were not included.

### Study Variables and Data Collection

Study variables included those identified as risk factors for development of CDAD, those associated with disease severity in previous studies, or those that logically predisposed patients to other severe disease outcomes. We collected patient demographic, historic, radiographic, and laboratory information.

Demographic variables included age, gender, hospital service (i.e., medical, surgical, obstetric, or gynecologic), date of positive *C. difficile* toxin sample, and length of hospitalization. We also collected number of antimicrobial drug classes used during hospitalization before positive *C. difficile* assay result, class and number of days used for each antimicrobial drug, and starting date and type of CDAD treatment initiated on or after the day the positive *C. difficile* toxin samples.

Historic data included use of corticosteroids, immunomodulating drugs, chemotherapy for hematologic and solid organ malignancies, proton pump inhibitors, and histamine-2 blockers. All medication data were limited to the 30 days before each patient’s first positive *C. difficile* toxin result. Physician-documented medical conditions included cardiovascular disease, diabetes mellitus, chronic kidney disease, past or current need for hemodialysis (not including intensive care unit [ICU] setting), pulmonary disease, hematologic malignancy, solid tumor malignancy, and immunocompromisation (i.e., solid organ or hematopoietic stem cell transplantation, immunoglobulin deficiencies, use of immunosuppressive agents, and severe autoimmune syndromes not associated with other malignancy).

Laboratory data were collected for a 7-day interval spanning 4 days before and 2 days after the day of submission of the first *C. difficile*–positive fecal specimen. This interval was chosen to account for variability in the promptness of *C. difficile* testing and initiation of treatment among healthcare providers. We recorded maximum leukocyte, serum glucose, creatinine, alanine aminotranferase levels, and minimum serum albumin concentrations.

Other variables were clinical or radiographic evidence of a small bowel obstruction or ileus in addition to abdominal and pelvic computed tomography (CT) scans with abnormal findings (colitis, pericolonic stranding, and abnormal rectal findings) documented anytime during hospitalization before laboratory diagnosis of CDAD. We also included skilled-nursing home or rehabilitation stays within 60 days before laboratory diagnosis of *C. difficile* infection, acute-care hospitalization within 30 days before diagnosis, date of death, date and number of admissions to ICU, number of surgical procedures, and enteral or total parenteral nutrition within 30 days before diagnosis of CDAD.

### Definition of Severe CDAD

Patients were defined as having severe CDAD if they met at least 1 of the following criteria: 1) death within 30 days after onset of symptoms or positive assay in which *C. difficile* infection was a major contributor; 2) >1 ICU admissions in which *C. difficile* infection was a major contributor; 3) colectomy or other surgery directly attributed to *C. difficile*; or 4) intestinal perforation in the presence of *C. difficile* infection. To minimize subjectivity, cases were reviewed independently by 2 study personnel directly involved with data collection and extraction and were counted as severe only if both reviewers agreed. A third investigator, who was not involved with data collection, reviewed each case and acted as a tie-breaker.

### Statistical Methods

The analysis was conducted in 3 stages. First, all-cause deaths and incidence of severe CDAD and death directly related to CDAD were explored in relation to age group. Second, univariate analyses were used to identify significant differences in variables for patients with severe and nonsevere disease. Chi-square testing with continuity correction was used to compare intergroup variation between nonparametric variables. Fisher exact tests were used if expected counts were <5. Mann-Whitney tests of ranked data were used to compare ordinal/parametric variables given the size discrepancy between the severe– and nonsevere–disease cohorts and to adjust for potential deviation from a normal distribution. Third, 2 logistic regression models were created. The first model was designed to evaluate independent associations between disease severity and antimicrobial drug use, demographics, and significant clinical variables identified from univariate analyses (prior nursing home/rehabilitation stays or acute-care hospitalizations, immunocompromisation, small bowel obstruction or ileus, and abnormal radiographic findings). The second model was designed to assess independent associations of laboratory variables with CDAD severity. Clinical variables likely to influence these laboratory values, such as hemodialysis, steroid use, and chemotherapy use, were included in this model. We used 2 models, rather than combining all variables into 1 model, because the small number of severe CDAD cases relative to total number of CDAD cases and large number of variables and covariates with potential collinearity in a combined model would decrease the power to detect statistical significance. Odds ratios (ORs) and 95% confidence intervals were calculated for each variable in the regression models (SPSS version 10; SPSS Inc., Chicago, IL, USA).

## Results

### Study Population

For the study interval, we identified 336 patients and 373 hospitalizations. However, to minimize the underestimation of variance among our sample population, we analyzed data from only 1 admission per patient (initial hospitalization), for a total of 336 hospitalizations.

The all-cause crude mortality rate during initial admissions was 10.1%. Most (82%) CDAD patients were >50 years of age; crude mortality rate in this group was 12.0%. For patients <50 years of age, crude mortality rate (1.7%) was markedly lower; for patients >70 years of age, crude mortality rate was highest (15.4%) (Table 1).

**Table 1:** All-cause deaths of inpatients with laboratory-confirmed CDAD, June 2005–May 2006*

Age group, y	Total no. patients	No. deaths	% Case-fatality†
18–50	60	1	1.7
51–60	70	7	10.0
61–70	76	6	7.6
71–80	83	12	14.5
81–90	40	7	17.5
>90	7	1	14.3
Total	336	34	10.1

*CDAD, *Clostridium difficile*–associated disease. †Percentage of deaths within age group.

The study definition for severe CDAD was met by 41 (12.2%) patients. Incidence of severe CDAD among all patients with CDAD was markedly higher in patients >70 years of age (p = 0.001). Of all patients, 21 (6.3%) died as a result of CDAD according to physician impression from chart review; none was <50 years of age (Table 2). Proportion of severe CDAD cases among patients with CDAD on these services did not differ significantly according to service (p = 0.18): 64% medical, 33% surgical, and 3% obstetric or gynecologic. Numbers of days from admission to laboratory diagnosis of CDAD patients with or without severe CDAD were similar (6.6 vs. 8.2; p = 0.13), as were lengths of hospitalization (18.3 vs. 18.2; p = 0.70).

**Table 2:** Severe CDAD and death as a result of CDAD, by age group, June 2005–May 2006*

Age group, y	Total no. patients	Severe CDAD, no. (%)	Deaths from CDAD, no. (%)
18–50	60	3 (5.0)	0 (0)
51–60	70	8 (11.4)	5 (7.1)
61–70	76	4 (5.2)	1 (1.3)
71–80	83	13 (15.7)	8 (9.6)
81–90	40	11 (27.5)	6 (15.0)
>90	7	2 (28.6)	1 (14.3)
Total	336	41 (12.2)	21 (6.3)

*CDAD, *Clostridium difficile*–associated disease.

### Univariate Analysis

Table 3 lists variables (except antimicrobial drug use) and laboratory values included in univariate analysis. Mean age of patients was 64 years. Patients with severe CDAD were significantly older (mean age 71 years) than those without severe disease (mean age 63 years); p = 0.001, Mann-Whitney test of ranked data. Proportion of male and female patients with or without severe CDAD did not differ significantly.

**Table 3:** Univariate analysis results for 336 patients with and without severe CDAD, June 2005–May 2006*

Variable	All patients, %†	Severe CDAD, % (n = 41)‡	Nonsevere CDAD, % (n = 295)	OR§	p value
Age >70 y	38.7	63.4	35.3	3.18	0.001¶
Female	48.2	51.2	47.8	1.15	0.807
Chemotherapy use§	16.1	9.8	16.9	0.53	0.343
Corticosteroid use§	25.6	31.7	24.7	1.41	0.444
Proton pump inhibitor use§	63.7	61.0	64.1	0.88	0.832
H2 blocker use	32.1	34.1	31.9	1.11	0.909
Enteral feeding	21.7	26.8	21.0	1.38	0.520
Parenteral feeding	3.3	2.4	3.4	0.71	1.000
Cardiovascular disease	41.7	53.7	40.0	1.74	0.135
Pulmonary disease	19.3	24.4	18.6	1.41	0.508
Diabetes	22.6	22.0	22.7	0.96	1.000
Renal disease	22.0	19.5	22.4	0.84	0.831
Hemodialysis	6.0	4.9	6.1	0.79	1.000
Immunocompromised	17.3	4.9	19.0	0.22	0.044¶
Malignancy	46.9	39.0	47.1	0.72	0.420
Small bowel obstruction or ileus	8.3	19.5	6.8	3.33	0.014¶
Abnormal abdominal CT scan	28.3	78.0	21.4	13.09	<0.001¶
Prior hospitalization	39.9	56.1	37.6	2.12	0.036¶
SNF/rehabilitation stay	22.9	36.6	21.1	2.17	0.043¶
Max glucose level >150 mg/dL	49.1	70.7	46.1	2.83	0.005¶
ALT >40 U/L	23.1	28.2	22.3	1.37	0.540
Min albumin level <2.5 g/dL	27.7	59.0	22.8	4.89	<0.001¶
Max creatinine level >2 mg/dL	22.0	41.5	19.3	2.96	0.003¶
Max leukocyte count >20,000/μL	28.3	53.7	24.7	3.52	<0.001¶
Mean max leukocyte count × 10^3^/μL	17.6	25.8	16.5	–	<0.001¶

*CDAD, *Clostridium difficile*–associated disease; OR, odds ratio; H2, histamine-2; CT, computed tomography; SNF, skilled-nursing facility; max, maximum; ALT, alanine aminotransferase; min, minimum. †Total of 336 patients were included in analysis except for ALT (n = 286) and albumin (n = 295). ‡n = 39 for ALT and albumin. §OR for severe CDAD in patients with positive *C. difficile* assay results; calculated for binary variables only (by χ^2^ or Fisher exact test) with exception of mean leukocyte count (significance calculated using Mann-Whitney test of ranked data). ¶Statistically significant at α = 0.05.

Other variables that did not differ significantly between patients with or without severe CDAD were underlying medical illness, malignancy, use of nonantimicrobial medications (including steroids and chemotherapy), and enteral or parenteral feeding (Table 3). CDAD was significantly less severe in patients who were immunocompromised or receiving immunosuppressive medications than in those who were not immunocompromised (OR 0.22, p = 0.044).

Other variables associated with severe disease included small bowel obstruction or ileus (OR 3.33, p = 0.014), abdominal CT results suggestive of colorectal pathologic changes (OR 13.09, p<0.001), acute-care hospitalization within 30 days before CDAD laboratory diagnosis (OR 2.12, p = 0.036), and rehabilitation or skilled-nursing facility stay within the 60 days before CDAD diagnosis (OR 2.17, p = 0.043). Maximum leukocyte count was significantly higher for patients with severe CDAD, and significantly more patients with severe disease had a maximum leukocyte count >20,000 cells/μL, minimum albumin level <2.5 g/dL, maximum glucose level >150 mg/dL, and serum creatinine level >2 mg/dL (Table 3).

Some exposure to antimicrobial drugs during the 30 days before laboratory diagnosis of CDAD was noted for ≈85% of patients. Exposure to, or number of, antimicrobial drugs did not differ significantly among patients with or without severe CDAD. In the severe and nonsevere CDAD cohorts, 85.4% of patients had used any antimicrobial drugs (p = 1.0). In addition, no significant differences were found between exposure to any of the following groups of antimicrobial drugs for patients with severe or nonsevere CDAD: fluoroquinolones; penicillin derivatives with or without β-lactamase inhibitor; aminoglycosides; clindamycin; first-generation cephalosporins; second- through fourth-generation cephalosporins; carbapenems; trimethoprim/sulfamethoxazole; intravenous vancomycin; systemic antifungal drugs. Use of oral or intravenous metronidazole before laboratory diagnosis of CDAD (p = 0.860) did not differ significantly.

Antimicrobial drugs (including oral and rectal vancomycin and metronidazole) for CDAD were given to 291 (86.7%) patients during their hospitalization. A higher percentage of patients with severe CDAD than without CDAD were treated with oral vancomycin (OR 8.27, p<0.001), rectal vancomycin (OR 20.35, p<0.001), or intravenous metronidazole (OR 4.2, p<0.001) on or after the day of laboratory diagnosis of *C. difficile* infection. There was no significant difference in frequency of severe outcomes among patients treated with or without oral metronidazole (OR 1.02, p = 1.0). All patients with severe CDAD were treated with at least 1 antimicrobial drug with activity against *C. difficile*.

### Regression Analyses

The following variables from the logistic regression model to identify covariates independently associated with severe CDAD were significant (p<0.05): age >70 years, ileus or small bowel obstruction, and abnormal abdominal CT image (Table 4). The following variables were not significantly associated with development of severe CDAD when adjusted for covariates: immunocompromisation status, prior acute-care hospitalization, and stay in a skilled-nursing facility. The independent association of laboratory values with severe CDAD was also investigated by using a single binary logistic regression model covariate adjusted with factors that would logically or historically influence each value. Maximum leukocyte count >20,000 cells/μL, maximum creatinine level >2 mg/dL, and minimum albumin level <2.5 g/dL were all independently associated with severe CDAD (p<0.05; Table 5).

**Table 4:** Binary logistic regression model to identify variables independently associated with severe CDAD, 336 patients*

Variable	OR	95% CI
Age >70 y	3.35†	1.48–7.57
Female	0.93	0.42–2.03
Antimicrobial-drug use	1.76	0.60–5.21
Malignancy	0.73	0.33–1.65
Immunocompromised	0.38	0.07–1.96
Small bowel obstruction or ileus	3.06‡	1.00–9.39
Abnormal abdominal CT scan	13.54†	5.72–32.07
Prior hospitalization	1.39	0.61–3.18
SNF/rehabilitation stay	1.11	0.46–2.68

*CDAD, *Clostridium difficile*–associated disease; OR, odds ratio; CI, confidence interval; CT, computed tomography; SNF, skilled-nursing facility. Constant included in analysis. †p<0.05. ‡p = 0.05.

**Table 5:** Binary logistic regression model to identify independent associations of laboratory values and pertinent variables with severe CDAD, 285 patients*

Variable	OR	95% CI
Age >70 y	3.24†	1.42–7.38
Female	1.16	0.51–2.62
Antimicrobial-drug use	1.04	0.35–3.12
Malignancy	0.90	0.37–2.18
Chemotherapy	1.02	0.27–3.92
Steroid use	1.13	0.48–2.68
Hemodialysis	0.5	0.8–3.01
Max leukocyte count >20,000/μL	2.77†	1.28–6.0
Max glucose level >150 mg/dL	1.46	0.63–3.43
ALT >40 U/L	1.47	0.58–3.69
Min albumin level <2.5 g/dL	3.44†	1.56–7.57
Max creatinine level >2 mg/dL	2.47†	1.04–5.88

*CDAD, *Clostridium difficile*–associated disease; OR, odds ratio; CI, confidence interval; max, maximum; ALT, alanine aminotransferase; min, minimum. Constant included in analysis. †p<0.05.

## Discussion

As would be expected in a general hospitalized population, advanced age was associated with all-cause mortality rates. Similarly, the odds of severe CDAD and death attributable to CDAD increased with age, especially for patients >70 years of age. Advanced age is known to be associated with CDAD, but whether age influences the severity of disease outcomes is in conflict in different publications (*3,**6**,**12*). For example, a study of 72 hospitalized patients with endoscopically proven pseudomembranous colitis showed advanced age to be associated with higher mortality rates, but age of patients who died of or survived after pseudomembranous colitis did not differ significantly (*12*). In contrast, Loo et al. noted a clear increase in the 30-day mortality rate in CDAD patients >80 years of age (*3*). In our study, no patient 18–50 years of age died as a result of CDAD, and advanced age was a significant risk factor for illness and death among patients with CDAD. Unlike age, gender was not associated with severe CDAD; this finding is similar to those of studies that investigated the role of gender on development, recurrence, or severity of CDAD (*12*,*15*). However, a large US study based on International Classification of Diseases, 9th revision, codes showed that CDAD case-fatality rate was higher for men than for women (*6*).

Because patients with CDAD are older and have more concurrent illness than the general population, effect of residence in long-term and acute-care facilities on the development and course of CDAD has generated interest (*13*,*16*–*18*). Our univariate analysis showed each of these variables to be associated with severe CDAD. However, when adjusted for age and concurrent illness, prior hospitalizations at long-term and acute-care facilities were not significantly associated with severe CDAD. On the basis of our regression model results, it is likely that the significance of prior hospitalizations (noted with univariate analysis) was the result of the more advanced age of patients who had had prior acute- or skilled-nursing facility hospitalizations and that age was the clinically important variable.

We found no association between malignancy or chemotherapy and severe CDAD. In contrast, Duberkke et al. found that 57% of patients who had undergone allogenic stem cell transplant had severe CDAD, although severity of disease was based on grade of diarrhea and colitis (*19*). The reason for the absence of association between immunosuppression due to malignancy or chemotherapy and severe CDAD found in our study is unclear, but it is possible that at this institution, which has a large oncology and solid-organ transplantation population, clinicians are more likely to order laboratory testing, implement precautions, and empirically initiate treatment earlier for immunosuppressed patients with suspected CDAD, thus avoiding severe sequelae. In addition, immunosuppressive medications have been associated with higher mortality rates in patients with CDAD who do or do not have fulminant colitis, but our univariate analysis results suggested that immunosuppressive comorbid conditions or use of immunomodulating agents (other than chemotherapy for malignancy) were protective against severe CDAD (*1*,*15*,*20*). However, this protective association was no longer noted when we adjusted for other factors.

Antimicrobial-drug use has been studied extensively with regard to development of CDAD and, to a lesser extent, severity and recurrence of disease (*3*,*12*,*14*,*21*–*25*). We found no association between severe CDAD and total number of antimicrobial drugs used, class of antimicrobial drug, and duration of exposure. In particular, use of antimicrobial drugs that are commonly associated with CDAD, including clindamycin and fluoroquinolones, did not differ among patients in whom severe CDAD did and did not develop. According to findings of previous studies, it is probable that our study population’s exposure to antimicrobial drugs was a risk factor for CDAD. However, antimicrobial-drug exposure did not appear to predispose patients with CDAD to severe disease. Recent data also suggest that patients who continue to receive antimicrobial-drug therapy without activity against CDAD while being treated for CDAD have a higher likelihood of CDAD treatment failure (*26*). Our study did not take into account whether patients continued to receive antimicrobial-drug therapy after laboratory diagnosis of CDAD. Antimicrobial-drug stewardship, however, has been shown to be useful in reducing CDAD rates (*27*).

Our univariate model showed aggressive treatment regimens for *C. difficile*, such as intravenous metronidazole and oral or rectal vancomycin, to be associated with worse outcomes. This finding is likely the result of our standard hospital practice to upgrade treatment of the sickest patients from oral metronidazole to oral vancomycin, intravenous metronidazole, rectal vancomycin, or a combination of these, so that exposure to these antimicrobial drugs was more likely to have been a surrogate marker of severe disease.

Laboratory markers such as leukocytosis, increased creatinine levels, and decreased albumin or globulin levels may correlate with poor outcome for patients with CDAD. Studies have yielded variable results, although multiple studies have shown that markedly increased leukocyte counts correlate with more severe disease (*1*,*12*,*15*,*20*). Similarly, our logistic regression model showed the following variables to be significantly correlated with severe CDAD: maximum leukocyte count >20,000/μL, minimum serum albumin level <2.5 g/dL, and maximum serum creatinine level >2 mg/dL. These laboratory values were adjusted for potential effects of concurrent underlying clinical conditions or treatments, such as hemodialysis, and use of steroids or chemotherapy. We also found some radiographic abnormalities to be associated with severe disease. On the basis of the results of our analyses, laboratory and imaging abnormalities may be useful for predictive modeling of severe outcomes from CDAD.

One major limitation of this study was our dependence on the date of laboratory diagnosis of CDAD to define disease onset. Clinical signs and symptoms (e.g., diarrhea, bloody feces, or abdominal pain) may have developed before the patient was tested for CDAD, and the date of laboratory diagnosis likely reflected the timing of physicians’ clinical suspicion for *C. difficile* infection rather than exact onset of symptoms. Our inability to reliably assess the presence of diarrhea may also have resulted in inclusion of some patients colonized with but not clinically ill from *C. difficile*. In addition, we found that several patients had been treated with oral vancomycin before laboratory diagnosis of CDAD and that severe disease was more likely to develop in patients treated before laboratory diagnosis. These patients were likely treated on grounds of clinical suspicion and may have had more aggressive onset and worse clinical markers for disease before laboratory diagnosis. We also did not evaluate whether cessation or continuation of antimicrobial drugs other than metronidazole and oral vancomycin affected progression to severe *C. difficile* infection.

As discussed previously, maximum and minimum laboratory values were collected for the time interval spanning the 4 days before and 2 days after the day of submission of the first *C. difficile*–positive specimen. We included the 2 days after laboratory diagnosis to account for variability in the timing of recognition and response to positive *C. difficile* assay results, but this fairly broad time interval limits to some extent our ability to evaluate the diagnostic utility of these laboratory values. Abnormal creatinine levels and leukocyte counts during this 7-day interval, for example, may have reflected the natural history and course of severe disease and skewed values higher for patients with severe CDAD. Finally, growing evidence indicates that BI-NAP 0127 causes more severe CDAD, and in our population bacterial subtype was likely an unrepresented predictor of severe disease.

The results of our study suggest that readily available clinical data, such as age and basic laboratory and radiology data, are correlated with severe CDAD outcomes. These findings suggest that clinicians may be able to gauge the risk for severe outcomes without individual genotyping. This ability is likely to be valuable in community as well as tertiary-care settings. Use of these markers for early identification of patients at high risk for severe disease may facilitate rapid implementation of aggressive medical and surgical CDAD therapy.
